# Clinical Bacteriology

**Published:** 1900-09

**Authors:** 


CLINICAL BACTERIOLOGY.—For Physicians and Students. By Dr. Ernst Levy, Professor in the University of Strasburg. 1 E. And Dr. Felix Klemperer, Private Docent in the University of Strasburg. 1 E. Second enlarged and revised edition. Authorized translation by August A. Eshner, M. D., Professor of Clinic Medicine in the Philadelphia Polyclinic; Physician to the Philadelphia Hospital, etc. Pp. 441. Price, $2.50. Philadelphia: W. B. Saunders, 925 Walnut Street. 1900.

This book is intended to teach bacteriology from the clinical point of view. There are many works on pathology, and on clinical diagnosis, but as far as the reviewer knows, this is the only book on clinical bacteriology, and it is intended to furnish the general practitioner with a working knowledge of the subject. All physicians can not become experts with the microscope, but the average physician nowadays must be familiar with all the aids to diagnosis in order to rationally apply measures and methods in the treatment and prophylaxis of disease. This work is splendidly adapted to meet the wants of the general practitioner. T. J. B.

